# Pregnancy coercion, correlates, and associated modern contraceptive use within a nationally representative sample of Ethiopian women

**DOI:** 10.1080/26410397.2022.2139891

**Published:** 2022-12-05

**Authors:** Shannon N Wood, Jessica L Dozier, Celia Karp, Selamawit Desta, Michele R Decker, Solomon Shiferaw, Assefa Seme, Robel Yirgu, Linnea A Zimmerman

**Affiliations:** a Assistant Scientist, Department of Population Family and Reproductive Health, Johns Hopkins Bloomberg School of Public Health, Baltimore, MD, USA.; b PhD Student, Department of Population Family and Reproductive Health, Johns Hopkins Bloomberg School of Public Health, Baltimore, MD, USA; c Assistant Scientist, Department of Population Family and Reproductive Health, Johns Hopkins Bloomberg School of Public Health, Baltimore, MD, USA; d Director of Survey Operations, Department of Population Family and Reproductive Health, Johns Hopkins Bloomberg School of Public Health, Baltimore, MD, USA; e Bloomberg Professor of American Health, Department of Population Family and Reproductive Health, Johns Hopkins Bloomberg School of Public Health, Baltimore, MD, USA; f Women’s Health and Rights Program Director, Center for Public Health & Human Rights, Johns Hopkins Bloomberg School of Public Health, Baltimore, MD, USA; g Joint Professor, Johns Hopkins School of Nursing, Baltimore, MD, USA; h Associate Professor, School of Public Health, Addis Ababa University, Addis Ababa, Ethiopia; i Assistant Professor, Department of Population Family and Reproductive Health, Johns Hopkins Bloomberg School of Public Health, Baltimore, MD, USA

**Keywords:** reproductive coercion, contraception, reproductive autonomy, Ethiopia

## Abstract

Partner-perpetrated pregnancy coercion inhibits women’s reproductive autonomy. However, few studies have quantified pregnancy coercion and its effects on women’s health within low- and middle-income countries. Among a national sample of Ethiopian women, this study aimed to: (1) assess the prevalence of past-year pregnancy coercion and explore regional differences; (2) identify correlates; (3) examine the relationship between pregnancy coercion and modern contraceptive use. Analyses utilise cross-sectional data from Performance Monitoring for Action (PMA)-Ethiopia, a nationally representative sample of females aged 15–49 conducted from October to November 2019. Past-year pregnancy coercion was assessed via five items and analysed dichotomously and categorically for severity. Among women in need of contraception, bivariate and multivariable logistic regression examined associations between variables of interest, per aim, accounting for sampling weights and clustering by enumeration area. Approximately 20% of Ethiopian women reported past-year pregnancy coercion (11.4% less severe; 8.6% more severe), ranging from 16% in Benishangul-Gumuz to 35% in Dire Dawa. Increasing parity was associated with decreased odds of pregnancy coercion. Among women in need of contraception, experience of pregnancy coercion was associated with a 32% decrease in odds of modern contraceptive use (aOR = 0.68; 95% CI: 0.53–0.89); when disaggregated by severity, odds decreased for most severe pregnancy coercion (aOR = 0.59; 95% CI = 0.41–0.83). Results indicate that partner-perpetrated pregnancy coercion is prevalent across diverse regions of Ethiopia, and most severe forms could interrupt recent gains in contraceptive coverage and progress to sexual and reproductive health and rights. Providers must be aware of potential contraceptive interference and address coercive influences during contraceptive counselling.

## Résumé

La coercition en matière de grossesse exercée par le partenaire inhibe l’autonomie reproductive des femmes. Pourtant, peu d’études ont quantifié ce phénomène et ses conséquences sur la santé des femmes dans les pays à revenu faible ou intermédiaire. Se fondant sur un échantillon national d’Éthiopiennes, cette étude visait à : 1) évaluer la prévalence de la coercition relative à la grossesse au cours de l’année précédente et explorer les différences régionales ; 2) identifier les corrélats ; 3) examiner la relation entre la coercition en matière de grossesse et l’emploi de contraceptifs modernes. Les analyses utilisent des données transversales tirées de l’enquête PMA-Éthiopie (Performance Monitoring for Action), auprès d’un échantillon représentatif au niveau national de femmes âgées de 15 à 49 ans, d’octobre à novembre 2019. La coercition exercée l’année précédente a été estimée avec cinq éléments et sa gravité a été évaluée de manière dichotomique et par catégorie. Parmi les femmes ayant besoin d’une contraception, une régression logistique binaire et multivariée a permis d’examiner les associations entre les variables d’intérêt, par but, en tenant compte des poids de l’échantillonnage et du regroupement par zone de recensement. Environ 20,0% des Éthiopiennes ont fait état d’une coercition relative à la grossesse l’année précédente (11,4% moins grave ; 8,6% plus grave), allant de 16% à Benishangul-Gumuz à 35% à Dire Dawa. Une parité croissante était associée à une probabilité moindre de coercition. Parmi les femmes ayant besoin d’une contraception, l’expérience de la coercition pendant la grossesse était associée à une diminution de 32% de la probabilité d’emploi d’une méthode moderne de contraception (rapports de cotes ajustés = 0,68 ; 95% IC : 0,53–0,89) ; lorsqu’elle était désagrégée par gravité, la probabilité diminuait pour les actes les plus graves de coercition (rapports de cotes ajustés = 0,59 ; 95% IC : 0,41–0,83). Les résultats indiquent que la coercition en matière de grossesse exercée par le partenaire est prévalente dans plusieurs régions éthiopiennes et que les formes les plus graves pourraient interrompre les récents progrès accomplis dans la couverture contraceptive. Les prestataires doivent être conscients des interférences potentielles avec la contraception et s’attaquer aux influences coercitives lorsqu’ils conseillent sur la contraception.

## Resumen

La coerción reproductiva perpetrada por la pareja inhibe la autonomía reproductiva de las mujeres; sin embargo, pocos estudios han cuantificado la coerción reproductiva y sus efectos en la salud de las mujeres en los países de bajos y medianos ingresos. En una muestra nacional de mujeres etíopes, este estudio tenía como objetivo: 1) evaluar la prevalencia de la coerción reproductiva en el último año y explorar las diferencias regionales; 2) identificar correlatos; 3) examinar la relación entre la coerción reproductiva y el uso de anticonceptivos modernos. Los análisis utilizaron datos transversales provenientes de *Performance Monitoring for Action* (PMA)-Etiopía, una muestra representativa a nivel nacional de mujeres de 15 a 49 años realizada entre octubre y noviembre de 2019. La coerción reproductiva en el último año fue evaluada vía cinco ítems y evaluada dicótoma y categóricamente por gravedad. Entre las mujeres que necesitaban anticoncepción, la regresión logística bivariada y multivariable examinó las asociaciones entre las variables de interés, por objetivo, teniendo en cuenta los pesos de muestreo y agrupamiento por zona de enumeración. Aproximadamente el 20.0% de las mujeres etíopes informaron sufrir coerción reproductiva en el último año (11.4% menos grave; 8.6% más grave), que varió del 16% en Benishangul-Gumuz al 35% en Dire Dawa. El aumento de la paridad se asoció con menor probabilidad de coerción reproductiva. Entre las mujeres que necesitaban anticoncepción, la experiencia de coerción reproductiva estaba asociada con una disminución del 32% de la probabilidad de uso de anticonceptivos modernos (RMa = 0.68; 95% IC: 0.53–0.89); al desglosarse por gravedad, la probabilidad disminuyó para la mayoría de los casos de coerción reproductiva grave (RMa = 0.59; 95% IC = 0.41–0.83). Los resultados indican que la coerción reproductiva perpetrada por la pareja es prevalente en diversas regiones de Etiopía, y las formas más graves podrían interrumpir los logros recientes en la cobertura anticonceptiva. Los prestadores de servicios deben ser conscientes de la posible interferencia con la anticoncepción y abordar las influencias coercitivas durante la consejería anticonceptiva.

## Introduction

Autonomy and self-determination are fundamental human rights; one avenue through which these rights translate into women’s sexual and reproductive health is via choice surrounding use or non-use of contraception.^1^ Additionally, violence against women is a human rights violation, as specified in 1993 in the Declaration on the Elimination of Violence Against Women, that is known to impact women’s autonomy and self-determination.^2^ Subsequent policies and global development goals have reaffirmed that both the elimination of violence against women and universal access to reproductive health services are critical to achieving gender equality.^3^ Key hindrances to targets for Sustainable Development Goal (SDG)-5, focused on gender equality and women’s empowerment, are brought to the fore with reproductive coercion (RC), or the interference in reproductive health decisions and behaviours.^4,5^ RC may include contraceptive sabotage (i.e. prohibiting use of contraceptive methods through direct tampering), condom manipulation (i.e. condom refusal or manipulation to decrease the efficacy of condoms), and indirect pregnancy pressures (i.e. threats or coercion).^5–11^ While the American College of Obstetrics and Gynecology (ACOG) recognises RC as a threat to women’s bodily autonomy, the definition does not indicate directionality or gender of the coercive partner;^12^ subsequent definitions and studies have almost exclusively examined male partners’ desire for more children and women seeking to avert pregnancy. RC infringes on reproductive rights, particularly autonomy and self-determination, given that it does not allow for free and informed decision-making surrounding contraception and fertility.^1^ Moreover, it has a long-term impact on women’s health and well-being, as recognised by mounting evidence linking RC to adverse reproductive, sexual, and mental health outcomes.^5,7,13–15^

The Reproductive Coercion Scale (RCS) is the most widely used and comprehensive measurement tool for quantifying RC.^7,16^ It examines two dimensions: pregnancy coercion and condom manipulation.^16^ To date, the majority of RC research using the RCS has been conducted in the United States, where both survivors and perpetrators of RC indicate discordant pregnancy intentions as a primary driver.^8,11^ This literature base further specifies that some women may be particularly vulnerable to RC, including less educated women and racial minorities.^9,17^ While this research has been critical in understanding vulnerabilities and highlighting the health effects of RC, including its impact on contraceptive use and subsequent unintended pregnancy,^7,9^ much less is known about RC’s consequences for women’s health in low- and middle-income countries (LMICs).

To date, few studies conducted in LMICs have aimed to quantify RC using the RCS, with prevalence widely ranging from 19% in Cote d’Ivoire^18^ to 82% among intimate partner violence (IPV) survivors in Nairobi, Kenya.^19^ These studies, conducted in northern India,^20^ Niger,^15,21^ Côte d’Ivoire,^18,22^ Kenya,^19,23^ and Ethiopia,^24^ highlight a range of outcomes associated with RC, including decreased contraceptive use,^20,24^ increased covert use of contraception,^15,25^ and increased subsequent unintended pregnancy^20^ and post-traumatic stress disorder.^18^ While this research highlights the negative health consequences arising from RC, there are limitations to consider. These studies were primarily conducted among highly specific sub-populations hypothesised to be at highest risk of RC, including IPV survivors^19^ and married adolescent girls and young women,^15^ or within the context of interventions;^18,20^ to date, no nationally representative studies have examined RC using components of the RCS. Comparability is further limited by measurement differences; the Niger,^15^ northern India,^20^ Côte d’Ivoire,^18^ and Ethiopia^24^ studies substantially adapted the RCS to the country context; the Ethiopia study further included lack of partner support for contraception as a component of RC, and additional actors that could restrict women’s reproductive autonomy, specifically family members, were also examined within the India and Cote d’Ivoire studies.^20,22^ Due to discrepancies in RC measures across studies and concerns surrounding generalizability, further research is needed within nationally representative populations to understand the prevalence of partner-perpetrated RC and identify women who may be at highest risk for experiencing RC.

Clarity on prevalence, determinants, and health impacts of RC are particularly valuable in settings where contraception is available to women, however, barriers to use may remain. In Ethiopia, approximately one-quarter of women use modern contraceptive methods and 13.9% of women have an unmet need for family planning.^26^ Approximately 25% of pregnancies are unintended, with women in Ethiopia reporting having one more child on average than they want (total fertility rate of 4.6 vs. wanted fertility rate of 3.6).^27^ Hindrances to contraceptive use and constraints on reproductive autonomy may be particularly poignant for Ethiopian women, given high national rates of early marriage and childbearing. Over half of women report being married by age 18^27^ and early marriage has been linked to decreased relationship quality;^28^ approximately 13% of women begin childbearing between the ages of 15–18.^27^ Further, only 60% of women have their demand for modern contraception satisfied, and of contraceptive users, 10% of women report that a provider or their partner chose their method.^26^ Women face immense pressure to conceive children,^29^ coupled with limited negotiation in sexual and reproductive decision-making.^29,30^ Moreover, gender and power disparities, as evidenced by high rates of child marriage, IPV, and female genital cutting,^27^ may impede women’s access to and use of contraception. While Ethiopia has made considerable progress in improving access to reproductive health services nationally, regional disparities persist among several key reproductive health outcomes;^31,32^ specifically, 16% of women in rural areas have an unmet need for contraception, compared to only 8% of urban women.^26^ There is further variation in access to reproductive health services across regions (i.e. health facilities are much more widely available in urban centres both within regions and in the capital of Addis Ababa), which is associated with differential use of contraceptive and maternal health services.^32,33^ Regional disparities are compounded with high rates of child marriage, IPV, and female genital mutilation.^27,31^ The percentage of women aged 15–19 who have begun childbearing ranges from 23% in Afar to 3% in Addis Ababa.^27^ Contextual data is necessary to understand reasons for contraceptive non-use and the potential role of the partner in method selection, uptake, and continuation in order to ensure human rights components of autonomy and self-determination are upheld within Ethiopia.

While initial studies in LMICs have been pivotal in situating research on RC within the global landscape on women’s reproductive autonomy, no studies to date have examined RC, particularly using the RCS, within a nationally representative survey. This study focused on one dimension of the RCS, pregnancy coercion, given immense pressure for women to conceive following marriage. Given low condom usage (<1%) within ongoing partnership in Ethiopia, we do not explore condom manipulation.^26^ Against this backdrop, we aimed to: (1) assess the prevalence of past-year pregnancy coercion among a nationally representative sample of Ethiopian women and explore regional differences; (2) identify sociodemographic characteristics associated with the experience of pregnancy coercion; (3) examine the relationship between pregnancy coercion and modern contraceptive use.

## Methods

### Study design

Data were collected under Performance Monitoring for Action (PMA)-Ethiopia, a five-year research partnership (2019–2023) between Addis Ababa University (AAU), The Ethiopian Federal Ministry of Health (FMoH), and Johns Hopkins Bloomberg School of Public Health (JHSPH). Procedures are described in-depth elsewhere.^34^ Briefly, PMA-Ethiopia generates timely cross-sectional and longitudinal data on reproductive, maternal, and newborn health indicators to inform national and regional government priorities and policies specific to maternal and newborn health. Data are collected at the female, household, and service delivery levels. The present analysis utilises cross-sectional data collected from October to November 2019.

The primary study aims to focus more broadly on measuring key coverage indicators of maternal, newborn, and contraceptive health services. While the focus of the survey is on use of health services, additional questions related to hypothesised barriers to use were also included. Given emergent RC research in sub-Saharan Africa detailing the potential for the partner to influence women’s contraceptive use,^15,19^ the study team, including the primary author who serves as a Senior Technical Advisor for PMA-Ethiopia on Gender, and with the consultation of the FMoH of Ethiopia, prioritised a limited number of RC measures. Due to the range of items already included in the surveys and to minimise respondent fatigue, space for additional items was limited and served as pilot data for potential expansion on gender-related issues, should these data be of interest to in-country stakeholders.

### Study procedures

National cross-sectional data included both household and female surveys. Following census and listing, 35 households were randomly selected within each enumeration area (EA); all randomly selected households within a selected EA were eligible for the household survey. Eligibility of women for the female survey was determined by their age and location at the time of the household survey. Inclusion criteria comprised females aged 15–49 years who lived within the selected household or slept in the selected household the night before and were willing and able to provide informed consent and/or assent; all eligible women within the household were eligible for female survey. Participants provided oral informed consent in accordance with National Ethical Review Guidelines specifying appropriate procedures for a largely non-literate population.

If eligibility criteria were met and consent was given, the female survey was immediately administered. Survey administration took approximately 30–60 minutes. All data were collected using mobile phones by trained resident enumerators (REs). All surveys were translated prior to interview and interviews conducted in local language of participant preference; for culturally diverse regions, local translators assisted with interviews. Trainings focused specifically on asking sensitive questions, including items on RC and IPV, and ensuring participant confidentiality. RC items were embedded within the questionnaire alongside other partner involvement in family planning items and REs were trained to monitor for privacy breaches; if the interview was interrupted, the RE was trained to stop the interview and only continue once auditory and visual privacy was ensured and if the participant was still willing to answer the remaining questions. All procedures were in line with best practices for research on violence against women^35^ and included facilitated referral to psychosocial support at local health centres. Study procedures are detailed further elsewhere.^34^ Institutional Review Board approval was obtained at JHSPH (IRB00009391; initial approval 14 March 2019; amendment approval 5 September 2019) and AAU (075/13/SPH; approved 20 March 2019). All participants provided verbal consent to participate.

### Analytic sample

RC items were asked exclusively to the 5605 women who were married or living with a partner at time of interview. Women were excluded from the analytical sample if they were missing RC data (*n* = 63), resulting in a final sample of 5542 women. For analyses examining associations with modern contraceptive use, women were additionally excluded if they were not in need of contraception at the time of survey (i.e. currently pregnant or wanting a child soon/now as assessed via the Demographic and Health Survey standard prospective pregnancy intention item^36^) for a sample of 3998 women.

### Measures

The primary dependent variable of interest was experience of past-year pregnancy coercion, measured via the five-item pregnancy coercion sub-scale from the RCS developed, validated, and refined in the United States.^7,10,16^ Specifically, all partnered women were asked

*In the past 12 months, has your husband/partner: 1) Told you not to use family planning? 2) Said he would leave you if you didn’t get pregnant? 3) Told you he would have a baby with someone else if you didn’t get pregnant? 4) Took away your family planning or kept you from going to the clinic? 5) Hurt you physically because you did not agree to get pregnant?*

Items from the condom manipulation sub-scale of the RCS were not included given low prevalence of condom use within ongoing partnerships in Ethiopia.^26^ Items were pilot tested using cognitive interviewing prior to survey implementation and no issues with comprehension and interpretation of items were indicated.

Psychometric analyses indicated one latent construct for pregnancy coercion (eigenvalue = 1.84; factor loadings > 0.4) and moderate reliability (Cronbach’s alpha = 0.69). Two summary variables were created for past-year pregnancy coercion: (1) a binary variable where affirmative response to any of the five items indicated experience of pregnancy coercion (any pregnancy coercion) and (2) a categorical variable for pregnancy coercion severity (no pregnancy coercion experience, less severe pregnancy coercion only (item 1 of the RCS), and more severe pregnancy coercion (items 2–5 of the RCS)). The categorical variable served to disentangle more vague behaviours from those hypothesised to be more harmful with specified coercive intent.

Sociodemographic variables explored as potential covariates for RC were chosen on a conceptual basis, and included urban/rural residence, age, marital status, age at marriage, education, pregnancy status, parity, religion, and polygyny. All covariates were examined in binary or categorical form, with small groups (*n* < 20) combined when possible to maximise statistical power. Specifically, religion was simplified to examine Orthodox, Muslim, Protestant, and “Other,” which included predominantly Catholic women. Pregnancy status utilised data on gestational age to assess RC by trimester given differential exposure risk over this time period; given the past-12-month time frame for RC, we would expect RC experience to decrease with increasing gestational age, given that a woman is no longer at risk for pregnancy coercion once she is pregnant.

The secondary outcome of interest, modern contraceptive use, was measured for all sexually active women using standard items.^36,37^ Specifically, women were asked, “are you or your partner currently doing something or using any method to delay or avoid getting pregnant.” Modern methods comprised hormonal and barrier methods, sterilisation, emergency contraception, lactational amenorrhoea method (LAM), and the standard days/cycle beads method.^38^

### Analysis

Descriptive statistics assessed the distribution of sociodemographic characteristics. Prevalence and 95% confidence intervals (CIs) of individual pregnancy coercion items and summary measures of past-year pregnancy coercion were calculated. For overall pregnancy coercion and pregnancy coercion severity, past-year prevalence and corresponding CIs were reported by region. Next, bivariate distributions of each dependent variable by sociodemographic characteristics were examined. Bivariate and multivariable logistic regression models were used to examine differences in pregnancy coercion experience (RC vs. no RC) by each sociodemographic characteristic; models were first run unadjusted and then adjusted for all other significant demographic characteristics from the bivariate models (*p* < .1) to examine independent effects. This process was repeated for pregnancy coercion severity using unadjusted and adjusted multinomial logistic regression models (more severe or less severe RC vs. no RC). Last, bivariate and multivariable logistic regression models were run separately to examine the association between each RC variable (any pregnancy coercion, categorical pregnancy coercion as a factor variable) and modern contraceptive use among women in need of contraception; adjusted analyses accounted for variables with *p* < .1 in unadjusted models. All analyses were conducted in STATA 16, with statistical significance set *a priori* at *p* < .05. We used the svy command to account for stratification during sample selection, clustering within enumeration areas, and the application of survey weights.

## Results

### Sample characteristics

Table 1 summarises the characteristics of women in the analytic sample. Nearly all women were married (97.2%), and approximately three-quarters were between 20 and 39 years old (74.4%) and resided within rural communities (72.9%). Roughly half of women never attended formal schooling (48.1%). While one in 10 respondents had no children (9.9%), about half (45.0%) had four or more children. Approximately 12% of respondents were pregnant at the time of survey. Women represented a range of religious affiliations, with the largest proportions identifying as Orthodox (43.0%) and Muslim (31.5%). Polygynous relationships were relatively uncommon, though 11% of women indicated that their partner had other wives.

**Table 1.** Sociodemographic characteristics of study sample (*n* = 5542), unweighted and weighted

Sociodemographic characteristic	Unweighted *n* (%)	Weighted *n* (%)
**Region**		
Addis Ababa	383 (6.9)	242 (4.4)
Tigray	593 (10.7)	296 (5.3)
Afar	338 (6.1)	71 (1.3)
Amhara	994 (17.9)	1281 (23.1)
Oromiya	1140 (20.6)	2190 (39.5)
SNNP	1072 (19.3)	1100 (19.8)
Somali	149 (2.7)	231 (4.2)
Benishangul-Gumuz	186 (3.4)	62 (1.1)
Gambella	250 (4.5)	21 (0.4)
Harari	231 (4.2)	24 (0.4)
Dire Dawa	206 (3.7)	26 (0.5)
**Residence**		
Urban	3562 (64.3)	1505 (27.2)
Rural	1980 (35.7)	4037 (72.9)
**Age**		
15–19	388 (7.0)	399 (7.2)
20–29	2327 (42.0)	2242 (40.5)
30–39	1897 (34.2)	1878 (33.9)
40–49	930 (16.8)	1022 (18.5)
**Marital status**		
Married	5377 (97.0)	5387 (97.2)
Living with a partner	165 (3.0)	155 (2.8)
Age at marriage*		
<15	999 (18.5)	1065 (19.7)
15–<18	1790 (33.1)	1892 (35.0)
≥18	2616 (48.4)	2448 (45.3)
**Education**		
Never attended	2494 (45.0)	2664 (48.1)
Primary	1857 (33.5)	1914 (34.6)
Secondary or higher	1187 (21.4)	960 (17.3)
**Pregnancy status**		
Not pregnant	4858 (87.7)	4867 (87.8)
0–3 Mo. pregnant	210 (3.8)	216 (3.9)
4–6 Mo. pregnant	255 (4.6)	239 (4.3)
7+ Mo. pregnant	219 (4.0)	221 (4.0)
**Parity**		
Nulliparous	616 (11.1)	548 (9.9)
1	1014 (18.3)	969 (17.5)
2–3	1631 (29.4)	1534 (27.7)
4+	2281 (41.2)	2491 (45.0)
**Religion**		
Orthodox	2426 (43.8)	2381 (43.0)
Muslim	1766 (31.9)	1745 (31.5)
Protestant	1210 (21.8)	1312 (23.7)
Other	140 (2.5)	105 (1.9)
**Husband/partner has other wives**	637 (11.5)	629 (11.4)

*Among *n* = 5405 women with complete age at marriage data.

### Prevalence of pregnancy coercion

Twenty per cent (95% CI: 17.9–22.3) of women reported any past-year pregnancy coercion, with less severe pregnancy coercion more common than severe forms (11.4% vs. 8.6%; Table 2). The most prevalent form of pregnancy coercion was the husband/partner telling the woman not to use family planning (17.1%; 95% CI: 15.1–19.3). Though relatively uncommon, approximately one out of every 100 women reported being hurt physically because they did not agree to get pregnant (0.9%; 95% CI: 0.6–1.4).

**Table 2.** Prevalence of pregnancy coercion (*n* = 5542), weighted

	% (95% CI)
*Individual items*: In the past 12 months, has your husband/partner:	
Told you not to use family planning?	17.1 (15.1–19.3)
Said he would leave you if you didn’t get pregnant?	5.3 (4.3–6.4)
Told you he would have a baby with someone else if you didn’t get pregnant?	4.4 (3.4–5.5)
Took away your family planning or kept you from going to the clinic?	4.2 (3.4–5.2)
Hurt you physically because you did not agree to get pregnant?	0.9 (0.6–1.4)
*Composite indicators*:	
Any experience of pregnancy coercion (items 1–5)	20.0 (17.9–22.3)
Pregnancy coercion severity	
Less severe pregnancy coercion only (item 1)	11.4 (9.8–13.2)
More severe pregnancy coercion (items 2–5)	8.6 (7.4–10.0)

**Figure 1.**
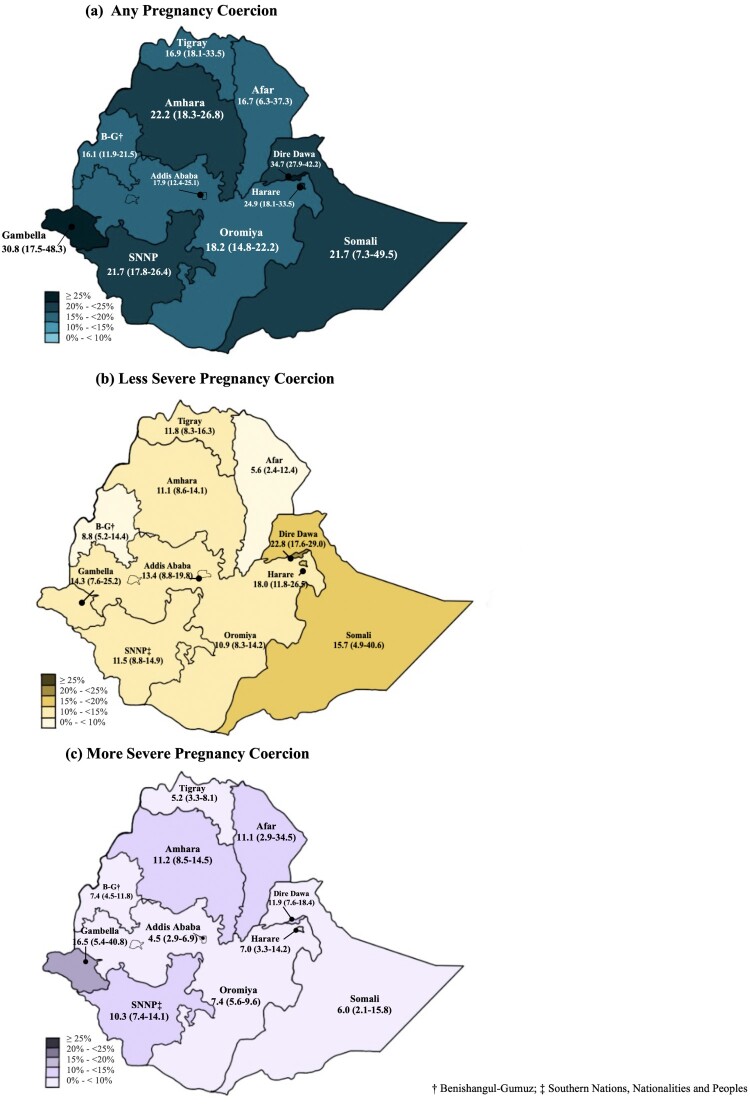
Prevalence of pregnancy coercion severity by region (95% confidence interval)

Regional variation of pregnancy coercion is shown in Figure 1. Prevalence of any pregnancy coercion ranged from 16% in Benishangul-Gumuz to 35% in Dire Dawa; less severe pregnancy coercion ranged from 5.6% in Afar to 22.8% in Dire Dawa and more severe ranged from 4.5% in Addis to 16.5% in Gambella. Less severe pregnancy coercion was generally more common than severe forms, however, this pattern did not hold for Afar, Amhara, and Gambella.

### Correlates of pregnancy coercion

Results of the bivariate and multivariable logistic regression models are shown in Table 3. Compared to Addis Ababa, residence in Dire Dawa was significantly associated with any pregnancy coercion (aOR = 2.56; 95% CI: 1.52–4.28). Increasing parity was associated with decreased odds of experiencing pregnancy coercion among women with one child (aOR = 0.69; 95% CI: 0.52–0.92), two to three children (aOR = 0.49; 95% CI: 0.37–0.63), and four or more children (aOR = 0.56; 95% CI: 0.43–0.74), compared to nulliparous women. Pregnancy status was associated with higher odds of experiencing pregnancy coercion in the unadjusted models, however, when adjusted, only 1–3 months gestation significantly increased odds of pregnancy coercion compared to non-pregnant women (aOR = 1.56; 95% CI 1.03–2.34). Other sociodemographic characteristics, including residence, age, marital status, education, religion, and polygyny, were not associated with any experience of past-year pregnancy coercion.

**Table 3.** Logistic and multinomial regressions of sociodemographic characteristics comparing pregnancy coercion vs. no pregnancy coercion (*n* = 5542), weighted

	Any pregnancy coercion			Pregnancy coercion severity					
				Less severe pregnancy coercion only			More severe pregnancy coercion		
	Row %	Unadjusted OR (95% CI)	Adjusted OR^†^ (95% CI)	Row %	Unadjusted RRR (95% CI)	Adjusted RRR^±^ (95% CI)	Row %	Unadjusted RR (95% CI)	Adjusted RRR^±^ (95% CI)
Region									
Addis Ababa	17.9	ref	ref	13.4	ref	ref	4.5	ref	ref
Tigray	16.9	0.93 (0.54–1.61)	1.00 (0.59–1.69)	11.8	0.87 (0.47–1.60)	1.18 (0.63–2.20)	5.2	1.14 (0.57–2.25)	0.87 (0.40–1.86)
Afar	16.7	0.92 (0.29–2.96)	0.96 (0.29–3.19)	5.6	0.41 (0.15–1.14)	0.57 (0.19–1.67)	11.1	2.43 (0.52–11.27)	1.54 (0.31–7.66)
Amhara	22.2	1.31 (0.80–2.15)	1.40 (0.86–2.28)	11.1	0.87 (0.50–1.52)	1.15 (0.62–2.12)	11.2	2.61 (1.47–4.67)***	1.90 (0.96–3.77)
Oromiya	18.2	1.02 (0.62–1.68)	1.10 (0.67–1.79)	10.9	0.81 (0.46–1.44)	1.12 (0.61–2.06)	7.4	1.64 (0.93–2.91)	1.16 (0.57–2.33)
SNNP	21.7	1.27 (0.77–2.10)	1.38 (0.85–2.26)	11.5	0.90 (0.51–1.58)	1.31 (0.73–2.37)	10.3	2.40 (1.31–4.39)**	1.65 (0.82–3.31)
Somali	21.7	1.27 (0.33–4.84)	1.36 (0.34–5.44)	15.7	1.23 (0.29–5.22)	1.67 (0.39–7.20)	6.0	1.39 (0.37–5.28)	1.00 (0.23–4.42)
Benishangul-Gumuz	16.1	0.88 (0.51–1.52)	0.91 (0.53–1.58)	8.8	0.64 (0.31–1.33)	0.77 (0.34–1.76)	7.4	1.60 (0.79–3.24)	1.20 (0.52–2.80)
Gambella	30.8	2.04 (0.86–4.81)	2.21 (0.92–5.31)	14.3	1.27 (0.56–2.88)	1.73 (0.74–4.03)	16.5	4.34 (1.14–16.55)*	3.58 (0.79–16.34)
Harari	25.0	1.53 (0.84–2.78)	1.57 (0.87–2.84)	18.0	1.47 (0.74–2.92)	1.71 (0.87–3.37)	7.0	1.70 (0.68–4.27)	1.31 (0.54–3.17)
Dire Dawa	34.7	2.44 (1.43–4.17)**	2.56 (1.52–4.28)***	22.8	2.14 (1.19–3.84)**	2.40 (1.40–4.14)**	11.9	3.34 (1.64–6.80)***	2.65 (1.17–6.00)*
Residence									
Rural	19.7	ref	ref	10.1	ref	ref	9.7	ref	ref
Urban	20.8	1.07 (0.79–1.44)	1.01 (0.72–1.40)	15.0	1.50 (1.06–2.13)*	1.53 (1.04–2.27)*	5.8	0.61 (0.41–0.90)*	0.64 (0.41–1.01)
Age									
15–19	22.9	1.10 (0.79, 1.53)	0.77 (0.54, 1.08)	11.7	0.96 (0.64, 1.44)	0.62 (0.39, 0.99)*	11.2	1.31 (0.84, 2.01)	0.84 (0.53, 1.32)
20–29	21.2	ref	ref	12.5	ref	ref	8.7	ref	ref
30–39	19.0	0.88 (0.73, 1.05)	1.02 (0.79, 1.31)	10.8	0.84 (0.67, 1.05)	1.09 (0.79, 1.50)	8.3	0.92 (0.70, 1.21)	0.86 (0.59, 1.27)
40–49	18.2	0.83 (0.66, 1.05)	0.99 (0.73, 1.34)	10.2	0.79 (0.58, 1.07)	1.05 (0.69, 1.60)	8.1	0.88 (0.63, 1.26)	0.79 (0.79, 1.29)
Marital status									
Married	19.9	ref	ref	11.4	ref	ref	8.4	ref	ref
Living with partner	25.4	1.38 (0.76–2.51)	1.22 (0.66–2.28)	10.0	0.94 (0.45–1.96)	0.73 (0.35–1.51)	15.4	1.97 (1.04–3.71)*	2.10 (1.12–3.91)*
Age at marriage^‡^									
<15	19.5	ref	ref	9.5	ref	ref	10.0	ref	ref
15–<18	19.7	1.01 (0.80, 1.28)	0.94 (0.74, 1.20)	10.8	1.14 (0.86, 1.53)	1.08 (0.81, 1.45)	8.8	0.88 (0.63, 1.22)	0.82 (0.58, 1.17)
≥18	20.4	1.06 (0.83, 1.35)	0.94 (0.73, 1.22)	12.5	1.34 (1.00, 1.78)*	1.18 (0.89, 1.56)	7.9	0.80 (0.56, 1.14)	0.80 (0.54, 1.20)
Education									
Never attended	19.6	ref	ref	10.5	ref	ref	9.2	ref	ref
Primary	21.6	1.13 (0.92–1.39)	0.99 (0.79–1.24)	12.1	1.19 (0.93–1.52)	0.93 (0.71–1.22)	9.5	1.06 (0.80–1.41)	1.02 (0.73–1.43)
Secondary+	18.0	0.90 (0.69–1.17)	0.71 (0.53–0.96)*	12.5	1.17 (0.86–1.60)	0.68 (0.48–0.97)*	5.5	0.58 (0.38–0.89)*	0.61 (0.36–1.04)
Pregnancy status									
Not pregnant	19.1	ref	ref	10.8	ref	ref	8.2	ref	ref
1–3 months pregnant	29.0	1.74 (1.17–2.58)**	1.56 (1.03–2.34)*	16.3	1.71 (1.05–2.79)*	1.58 (0.95–2.64)	12.8	1.77 (1.05–2.98)*	1.63 (0.95–2.80)
4–6 months pregnant	27.9	1.64 (1.09–2.46)**	1.44 (0.96–2.19)	16.5	1.71 (1.02–2.87)*	1.51 (0.88–2.60)	11.3	1.55 (0.94–2.53)	1.36 (0.82–2.24)
7+ months pregnant	24.0	1.34 (0.95–1.90)	1.24 (0.88–1.76)	13.6	1.34 (0.85–2.12)	1.24 (0.79–1.93)	10.4	1.34 (0.83–2.16)	1.22 (0.75–2.01)
Parity									
Nulliparous	30.9	ref	ref	19.4	ref	ref	11.6	ref	ref
1	22.2	0.64 (0.48–0.85)**	0.69 (0.52–0.92)**	13.2	0.60 (0.44–0.83)**	0.60 (0.43–0.83)**	9.1	0.70 (0.46–1.07)	0.68 (0.44–1.05)
2–3	16.9	0.45 (0.35–0.59)***	0.49 (0.37–0.63)***	10.2	0.44 (0.32–0.60)***	0.43 (0.31–0.59)***	6.7	0.48 (0.34–0.70)***	0.41 (0.30–0.64)***
4+	18.7	0.51 (0.39–0.67)***	0.56 (0.43–0.74)***	9.7	0.43 (0.31–0.58)***	0.43 (0.30–0.61)***	9.0	0.66 (0.46–0.94)*	0.52 (0.34–0.79)**
Religion									
Orthodox	19.8	ref	ref	11.2	ref	ref	8.6	ref	ref
Muslim	20.2	1.02 (0.73–1.43)	1.03 (0.74–1.44)	11.5	1.03 (0.68–1.54)	1.14 (0.76–1.72)	8.7	1.02 (0.68–1.51)	0.93 (0.61–1.41)
Protestant	19.7	1.00 (0.74–1.34)	1.00 (0.74–1.33)	11.3	1.01 (0.70–1.45)	1.13 (0.79–1.62)	8.4	0.98 (0.64–1.48)	0.92 (0.60–1.41)
Other	26.3	1.45 (0.81–2.59)	1.50 (0.83–2.72)	17.0	1.65 (0.86–3.19)	1.94 (0.99–3.80)*	9.3	1.18 (0.45–3.05)	1.18 (0.45–3.11)
Husband/partner has other wives									
No	20.1	ref	ref	11.9	ref	ref	8.3	ref	ref
Yes	19.2	0.94 (0.71–1.26)	1.01 (0.76–1.34)	7.9	0.66 (0.46–0.94)*	0.72 (0.51–1.02)	11.3	1.35 (0.96–1.90)	1.31 (0.93–1.86)

^†^ Logistic regression adjusted for pregnancy status and parity; ^±^Multinomial regression adjusted for residence, marital status, education, pregnancy status, parity, polygyny.

^‡^ Among *n* = 5405 women with complete age at marriage data.

*p-Value < .05; **p-value < .01; ***p-value < .001; bolded text indicates statistical significance at *p* < .05 level.

Results of the unadjusted and adjusted multinominal logistic regression models are also shown in Table 3. Compared to women in Addis Ababa, residence in Dire Dawa was associated with increased risk for less severe pregnancy coercion (versus no pregnancy coercion) (aRRR = 2.40; 95% CI = 1.40–4.14), whereas residence in Amhara (RRR = 2.61; 95% CI = 1.47–4.67), SNNP (RRR = 2.40; 95% CI = 1.31–4.39), Gambella (RRR = 4.34; 95% CI = 1.14–16.55), and Dire Dawa (RRR = 3.34; 95% CI = 1.64–6.80) were associated with increased risk of more severe forms of pregnancy coercion (versus no pregnancy coercion) in unadjusted models; all but Dire Dawa associations attenuated with adjustment. Urban residence was associated with increased risk of less severe pregnancy coercion (aRRR = 1.53; 95% CI = 1.04–2.27), but was marginally protective for more severe forms (aRRR = 0.64; 95% CI = 0.41–1.01). Age was significantly associated with decreased risk of less severe pregnancy coercion for women ages 15–19 years old only, as compared to 20–29-year-olds (aRRR = 0.62; 95% CI = 0.39–0.99), whereas living with a partner was associated with increased risk of more severe pregnancy coercion (aRRR = 2.10; 95% CI = 1.12–3.91). Compared to nulliparous women, increasing parity displayed protective associations for both less and more severe forms of pregnancy coercion across almost every category. In adjusted models, “Other” religion, comprising primarily Catholic participants, as compared to Orthodox, was associated with increased risk of less severe pregnancy coercion (aRRR = 1.94; 95% CI = 0.99–3.80); this association was not observed for more severe pregnancy coercion. Polygynous relationships were associated with decreased risk of less severe pregnancy coercion in unadjusted models, however, this association attenuated with adjustment.

### Association with modern contraceptive use

Among women in need of contraception, any experience of past-year pregnancy coercion was associated with a 32% decrease in the odds of modern contraceptive use (aOR = 0.68; 95% CI: 0.53–0.89; Table 4). When examined by severity of pregnancy coercion, odds of contraceptive use only significantly decreased for women who experienced the most severe forms of pregnancy coercion (aOR = 0.59; 95% CI = 0.41–0.83).

**Table 4.** Logistic regression of modern contraceptive use vs. no use by pregnancy coercion experience, among women in need of contraception^†^ (*n* = 3998), weighted

	Contraceptive use (%)	Unadjusted OR (95% CI)	Adjusted OR (95% CI)
Any pregnancy coercion^‡^			
No	48.3	ref	ref
Yes	39.3	0.69 (0.53–0.90)**	0.68 (0.53–0.89)**
Severity of pregnancy coercion^±^			
None	48.3	ref	ref
Less severe	42.8	0.80 (0.57–1.13)	0.77 (0.55–1.08)
More severe	34.7	0.57 (0.41–0.80)***	0.59 (0.41–0.83)**

^†^ Women in union who are not currently pregnant and do not want to have a child now soon/now.

^‡^ Adjusted model includes parity.

^±^ Adjusted model includes residence, marital status, education, parity, polygyny.

## Discussion

This manuscript presents the first nationally representative prevalence estimates of pregnancy coercion, where we found that one in five women in Ethiopia experienced pregnancy coercion in the past year and that less severe forms were more common than severe forms (11.4% vs. 8.6%). Moreover, we found substantial regional variation across Ethiopia (ranging from 16% to 35%), however, these regional differences were inconsistent when examined by pregnancy coercion severity. Results also indicate that some subgroups may be at increased risk of pregnancy coercion; specifically, nulliparous women appear to be at higher risk of experiencing pregnancy coercion than women with at least one child. Past-year pregnancy coercion decreased odds of modern contraceptive use by 32%, after accounting for sociodemographic characteristics. Decreased contraceptive use was most pronounced for women experiencing the most severe forms of pregnancy coercion (41% reduction). Below, we discuss each of these findings in turn.

Past-year prevalence of pregnancy coercion greater than 30% in Gambella and Dire Dawa warrants further qualitative exploration and continued quantitative investigation with larger sample sizes to understand sociocultural influences that may increase RC vulnerability and perpetration. Due to the significant dearth of research in the area of RC, it is not possible to compare these findings with other similarly designed studies. We note, however, that these findings are somewhat inconsistent with patterns of women’s empowerment as measured in the Demographic and Health Surveys (DHS), as neither region has systematically high or low levels of women’s decision-making regarding household decisions or decisions surrounding marriage and childbearing.^27^ This discrepancy underscores that RC is likely to operate separately from other indicators of women’s empowerment and calls for further research into the relationships between domains of empowerment, reproductive decision-making, and the underlying norms that may support the practice of RC; it is possible that RC does not track with empowerment given women’s internalisation of childbearing norms (i.e. less empowered women are more likely to accept their partner’s childbearing desires and therefore less likely to report RC experiences). Further, regional patterns in RC do not fully track with regional variation in contraceptive prevalence, with lowest contraceptive prevalence noted for Afar and Somali regions,^27^ although these regions were approximately equivalent with the national prevalence of RC (16.7 and 21.7%, respectively); such results allude to additional barriers impeding women’s contraceptive use within these contexts. Moreover, our results on regional differences were inconsistent when examined by pregnancy coercion severity, indicating a need to disaggregate RC forms to understand contributors to forms of RC that may have greater autonomy, and in turn, health, well-being, and human rights implications for women.

Disaggregation by severity allows for a more nuanced understanding of correlates of specific forms of pregnancy coercion. Living in an urban area was associated with increased risk of less severe pregnancy coercion, whereas it was associated with decreased risk of more severe forms. Similar opposing patterns by severity were observed for age, marital status, and polygyny. Several pathways may drive these differences and point to key distinctions in types of pregnancy coercion; further research is necessary to understand drivers of pregnancy coercion and how these intentions are tied to specific behaviours. Specifically, partners’ involvement in contraceptive decision-making, including warning against contraceptive use (i.e. less severe pregnancy coercion), may be acceptable in more egalitarian partnerships, which may be more widespread in urban settings, while explicit interference and sabotage may be less acceptable among urban couples. This hypothesis is consistent with trends in IPV behaviours in Ethiopia, which are more common in rural than urban areas.^27^ In situations of explicit interference and sabotage, women’s sexual and reproductive rights are severely infringed upon, as they are not able to fully exercise their preferences surrounding contraceptive decision-making.

Conversely, partners’ verbal discouragement of contraception may be enacted without coercive intent. Qualitative research indicates that while some partners may indeed persuade against contraceptive use for controlling purposes, others are genuinely concerned with contraceptive-related side effects.^19,29,39^ Importantly, this research highlights the need to understand less severe forms of pregnancy coercion, specifically partners’ verbal discouragement of contraception, given that 17.1% of women indicated their husband/partner told them not to use family planning in the past year. While this less severe form of pregnancy coercion was not significantly associated with decreases in modern contraceptive use, partner discouragement of family planning, either motivated by concern or control, can still impact contraceptive behaviour, as evidenced by prior research indicating increases in covert contraceptive use^15,25^ and more limited contraceptive method options^25^ for women experiencing RC. While some women may succeed in using contraception despite their partner’s discouragement – at least until faced with more severe forms of RC – they may not have full control over what methods they use; notably, inhibiting method choice infringes on women’s autonomy and self-determination through limiting free and informed choice. Future research should seek to understand the motivation behind partner discouragement and the ways in which women negotiate with partners who discourage contraceptive use in order to maximise their own reproductive autonomy. Notably, recent work in Niger aimed to increase specificity of this less severe item by separating it into two items, including (1) “made her feel bad or treated her badly for wanting to use a family planning method to delay or prevent pregnancy”; and (2) “tried to force or pressure her to become pregnant.”^15,21^ Future research within PMA-Ethiopia will similarly use this wording and continue to refine RC measurement.

High pregnancy coercion prevalence and higher odds of pregnancy coercion experience among nulliparous women are largely consistent with the limited RC literature in sub-Saharan Africa, namely, Niger and Nairobi, which highlight the immense pressure that women face to begin childbearing.^15,19^ Further, these results are compatible with qualitative literature that highlights the complexities women face to achieve their childbearing goals, including the extent to which they will go to use contraception covertly when faced with discordant fertility intentions from their partner.^29,39^ While women’s empowerment and reproductive health programmes aim to bolster women’s health and well-being through increased educational attainment and employment, often resulting in delayed childbearing, they must also consider the potential repercussions of these efforts, including compromised safety. Such programmes must also address harmful gender norms at the community level and equip men with skills to become supportive partners and fathers; these interventions are imperative to ensuring women’s reproductive rights are upheld.

This analysis is not without limitations. Foremost, the cross-sectional design limits conclusions surrounding temporality. Future work aims to overcome these limitations to further disentangle pathways between RC and reproductive health outcomes, including contraceptive use, as well as examine predictors of RC. While study authors were involved in data collection for the parent study, the primary study objectives were not focused on RC and gender and power dynamics; as such, formative work to adapt the RCS to the Ethiopian context was not undertaken and partner covariate data were limited. Qualitative data are needed to contextualise results and understand potential adaptation needs of the RCS. As noted previously, the first item of the RCS indicated a markedly high prevalence compared to other RCS items, warranting stratification by pregnancy coercion severity. While this measure of severity is imperfect, given that we did not ask about frequency of each act, it did allow for a more nuanced measurement of pregnancy coercion and examination of partners’ discouragement of family planning use. We encourage future research to seek to understand partners’ motivations for persuading against contraceptive use and whether these behaviours are motivated by concern or control, as well as aim to assess frequency of each behaviour for a better calibration of pregnancy coercion severity.

Strengths include a national sample and a nuanced measure of pregnancy coercion severity. This provided us with a large enough sample to disentangle covariates of less and more severe pregnancy coercion and understand each measure’s relationship to contraceptive use. With the exception of the Nairobi study among IPV survivors,^19^ which utilised a continuous metric of RC given the high underlying violence prevalence, all studies to date have examined RC as a binary measure. Large-scale surveys, such as PMA and DHS, are encouraged to use more specific RC items and continue to disaggregate RC severity in order to better understand coercive partner dynamics that may hinder women’s reproductive health and rights. National and cross-national estimates can not only help understand the magnitude of this abuse in LMICs, but also establish pathways between RC and health outcomes; such research is necessary to ensure gender equity and uphold commitments to women’s health and rights. Longitudinal designs are needed to understand the longer-term impact of RC on women’s health, and cascading social, financial, educational, and well-being outcomes.

## Conclusions

This research indicates that pregnancy coercion is prevalent throughout diverse regions of Ethiopia. To enact long-term changes in social norms that have the potential to reduce RC, programmes must engage boys and men to combat harmful beliefs around contraception and reproduction. This research makes it clear that childbearing is not only a woman’s issue, and men’s opinions surrounding women’s fertility and contraceptive use play a central role in shaping women’s reproductive autonomy. In the shorter term, national training programmes for family planning providers should include education on the potential role of partner interference in women’s contraceptive use and help providers disentangle partner concern versus coercive intent. While recognising that joint couple decision-making on reproduction and childbearing is the ideal, this research acknowledges that this standard is not always achievable within couples due to gender and power imbalances. To protect women’s health and rights and to ensure that her autonomy and self-determination are maximised, ultimately, a woman should have the final authority in deciding if and when to have children, free from violence or coercive influence, to improve her own health and the well-being of her family and community.

## Data Availability

Data are available by request at www.pmadata.org.
